# Combined intervention program of serious virtual reality games focused on controllability and Stress Inoculation Training in patients with major depression

**DOI:** 10.3389/fpsyt.2026.1701803

**Published:** 2026-05-08

**Authors:** Hesed Virto-Farfan, Micaela Manchego, Jakeline Jayo, Gustavo E. Tafet

**Affiliations:** 1 Neuroscience Research Circle (CIN), Universidad Andina del Cusco, Cusco-Perú, Peru; 2 Department of Psychiatry and Behavioral Sciences, Texas A&M University, College Station, TX, United States

**Keywords:** depression, Stress Inoculation Training, virtual reality, virtual reality exposure therapy, VR

## Abstract

**Introduction:**

Major depressive disorder (MDD) is a debilitating mental health condition with a high global prevalence, characterized by profound psychological and neurobiological alterations. Although many patients respond effectively to pharmacological and psychotherapeutic interventions, the persistently high rate of treatment resistance underscores the urgent need for novel strategies that target stress regulation and promote neuroplasticity. The aim of this study was determining the effect of a Virtual Reality Stress Inoculation Training (VR-SIT) in patients with MDD suffering mild and moderate episodes. In this experimental study, 54 patients diagnosed with MDD were randomly assigned to either the VR−SIT group or the Traditional Treatment (TT) group. Inclusion criteria included a diagnosis of MDD and a Hamilton Depression Scale score greater than 15. The TT group received standard pharmacological and psychotherapy treatment, while the VR−SIT group received pharmacological treatment combined with a virtual reality intervention. Depression severity and motivation were assessed using the Hamilton Depression Rating Scale (HAM-D) and a visual analog scale, respectively.

**Results:**

The mean age of participants was 26.26 years, with a mean disease duration of 9.93 months. Both groups exhibited a significant reduction in Hamilton Depression Scale scores from 18.15 pre−treatment to 10.33 post−treatment with no significant differences between groups. However, significant increases in motivation were observed in the VR−SIT group during Week 3 and at the end of the intervention, indicating higher motivation levels compared to the TT group.

**Conclusions:**

Both TT and VR−SIT interventions significantly reduced depressive symptoms; however, only VR−SIT was associated with a progressive and significantly greater increase in motivation. Future research should investigate long−term effects, elucidate the specific mechanisms underlying the controllability component, and standardize treatment protocols to enhance comparability and robustness.

## Introduction

Major depressive disorder (MDD) is a prevalent and disabling mental health condition characterized by persistent low mood, anhedonia, and cognitive and somatic symptoms that substantially impair daily functioning (1). Globally, MDD affects approximately 280 million individuals and is associated with a lifetime prevalence of 10–15%, high rates of treatment resistance, and significant neurobiological alterations, resulting in considerable personal, social, and economic burden (2). Despite the availability of pharmacological and psychotherapeutic interventions, a substantial proportion of patients fail to achieve adequate symptom remission, highlighting the need for innovative approaches that more effectively target stress regulation and neuroplastic mechanisms (3).

Stress-related neurobiological dysregulation plays a central role in the pathophysiology of MDD. The hypothalamic–pituitary–adrenal (HPA) axis is a key mediator of stress responses, with daily stressors activating limbic structures such as the amygdala and initiating corticotropin-releasing factor (CRF) release from the hypothalamus. This cascade stimulates adrenocorticotropic hormone (ACTH) secretion and subsequent cortisol release from the adrenal cortex (4, 5). Persistent or dysregulated activation of this system contributes to emotional dysregulation, cognitive impairment, and vulnerability to depressive symptomatology.

Stress represents a universal adaptive response to perceived threats to physical or psychological integrity (4, 5). Classical formulations of stress have conceptualized it as a dynamic biological process encompassing alarm, resistance, and exhaustion phases (4, 5). At the neural level, acute stress modulates inhibitory and excitatory circuits, notably through alterations in GABAergic activity within the hippocampus and prefrontal cortex, influencing limbic system balance and behavior (6, 7). While such responses are essential for short-term survival, sustained stress exposure increases allostatic load, inflammation, and excitotoxic vulnerability, particularly in the context of adverse social and environmental conditions (8).

Importantly, stress is not inherently pathological. Under controlled conditions, moderate stress can promote adaptive neuroplastic changes, including synaptic remodeling and increased brain-derived neurotrophic factor (BDNF) signaling, thereby supporting learning, memory consolidation, and resilience (5, 9). This concept of “eustress” emphasizes the potential benefits of manageable challenges that mobilize physiological resources without overwhelming adaptive capacity (9). The interaction between genetic susceptibility, epigenetic modulation, and environmental exposure determines whether stress responses become maladaptive or resilience-enhancing, underscoring the importance of timely, structured interventions (10). Consistent with this framework, elevated cortisol levels have been reported in MDD and related affective disorders, reflecting altered stress regulation across diverse populations (11).

Advances in digital mental health technologies have expanded opportunities for stress assessment and intervention (12, 13). Within this context, virtual reality (VR) has emerged as a promising therapeutic tool, allowing the simulation of ecologically valid, stress-inducing scenarios within controlled and safe environments (13, 14). VR-based serious games further enhance this potential by integrating therapeutic objectives with interactive and motivational game mechanics, fostering experiential learning and treatment adherence (12).

VR interventions that emphasize controllability are particularly relevant for MDD, a condition frequently characterized by perceived helplessness and diminished agency. By enabling users to exert control over virtual environments, controllability-focused VR interventions may enhance coping skills and emotional regulation (14, 51). This approach aligns closely with Stress Inoculation Training (SIT), a cognitive-behavioral framework designed to prepare individuals to manage stress through skill acquisition and adaptive appraisal (15). SIT has demonstrated benefits in reducing anxiety-related distress and strengthening coping and self-efficacy across applied settings (16–21).

Stress Inoculation Training (SIT) is a cognitive-behavioral intervention originally developed by Meichenbaum to enhance stress resilience by strengthening coping repertoires, adaptive appraisal, and perceived controllability when facing stressors (15). SIT is typically described as a structured, phased approach comprising: (i) conceptualization or education, in which individuals develop a coherent understanding of stress responses and identify maladaptive appraisals; (ii) skills acquisition and rehearsal, in which coping strategies such as cognitive restructuring, relaxation, problem-solving, and self-instruction are trained and practiced; and (iii) application and follow-through, in which these skills are systematically implemented and generalized to progressively more demanding stress contexts (15). Across applied settings, SIT has shown benefits in reducing psychological distress and improving adaptive functioning, with evidence of efficacy in reducing anxiety-related outcomes and enhancing performance under stress (16–21). Importantly, SIT’s emphasis on coping self-efficacy and perceived controllability provides a direct theoretical pathway through which stress-focused training may also influence motivational processes and depressive symptom severity, particularly in conditions characterized by helplessness and diminished agency (15, 16).

In the present trial, the comparison was between a conventional (non-VR) delivery format and a VR-based delivery format of the same stress-focused training framework. Specifically, the Traditional Treatment (TT) condition corresponded to a structured face-to-face stress-management intervention delivered within routine community mental health care, including psychologist-led sessions and therapeutic accompaniment by trained nursing staff, but implemented without virtual reality environments (15). The VR-SIT condition preserved the same therapeutic logic and skill targets but delivered the application and practice components through immersive, controllability-oriented virtual scenarios designed to facilitate experiential learning, graded exposure to stressors, and participant engagement (12, 14, 51). Accordingly, the primary difference between groups was the delivery modality (conventional versus immersive VR), rather than differences in the underlying intervention rationale, allowing the study to specifically examine whether VR-based implementation confers incremental benefits on motivational engagement and depressive symptom severity beyond a non-VR SIT-based approach (15, 16). Digital and hybrid adaptations of SIT, including manualized and computer-based formats, have demonstrated feasibility and efficacy in high-stress populations such as military personnel, students, and individuals with medical or psychological vulnerability (17–21). These findings support SIT as a flexible, scalable intervention model capable of addressing both psychological and somatic dimensions of stress-related disorders.

The integration of SIT with VR technology capitalizes on the immersive, interactive properties of VR to enhance skill generalization and experiential learning. VR-based SIT enables graded exposure to stressors and repeated practice of coping strategies in realistic yet controlled contexts, facilitating transfer to real-world situations (14, 22, 23). This approach has shown particular utility in stress-related conditions such as PTSD, where VR allows systematic modulation of stress intensity while maintaining patient safety (23, 24). More broadly, VR has demonstrated promise across multiple psychotherapeutic modalities, including exposure therapy, cognitive-behavioral therapy, and mindfulness-based interventions for depression (25–27), with growing evidence supporting its potential to reduce depressive symptoms and improve well-being (28–30).

The present study hypothesized that Virtual Reality Stress Inoculation Training (VR-SIT) would reduce depressive symptom severity in individuals with mild to moderate MDD by enhancing stress regulation, self-efficacy, and emotional control through immersive, interactive simulations. It was further hypothesized that VR-SIT would improve adherence and therapeutic engagement compared with conventional approaches, given the motivational and experiential advantages of VR-based interventions. Accordingly, the aim of this study was to determine the effect of a VR-SIT intervention in patients with mild and moderate episodes of Major Depressive Disorder.

## Materials and methods

### Design

The present study had a randomized pilot experimental design, with two parallel intervention groups.

### Sample

Participants for this study were individuals diagnosed with mild and moderate major Depressive Disorder (MDD), meeting the diagnostic criteria outlined in the DSM-5 or CIE-10. The sample was divided into a control group and an experimental group. Participants were recruited from the Community Mental Health Center of San Sebastian.

Inclusion criteria included the willingness to participate in the study, mild or moderate major depression diagnosed by a psychiatrist in the last week, no pharmacological treatment initiated at the time of the baseline evaluation, no other severe psychiatric disorders (such as bipolar) based on self-report, age over 18 years old, and agree to participate during the 3 weeks. Exclusion criteria included inactive and irregular attendance, unwillingness to continue attending practices, other severe rheumatological, endocrinological, or neurological illness and migration, prior exposure to Virtual Reality (VR) interventions for mental health treatment.

The sample consisted of participants meeting the eligibility criteria to enter the study during the experiment. A total of 96 individuals who obtained the necessary scores based on the relevant questionnaires were enrolled through the convenience sampling method. From this initial group, a total of 54 patients diagnosed with MDD were randomly assigned to either the VR−SIT group or the Traditional Treatment (TT) group. Inclusion criteria included age over 18, a recent diagnosis of MDD, and a Hamilton Depression Scale score greater than 15. The TT group received standard pharmacological and psychotherapy treatment, while the VR−SIT group received pharmacological treatment combined with a virtual reality intervention. Depression severity and motivation were assessed using the Hamilton Depression Scale and a visual analog scale, respectively. The study was conducted in accordance with the Declaration of Helsinki.

**Figure 1:**
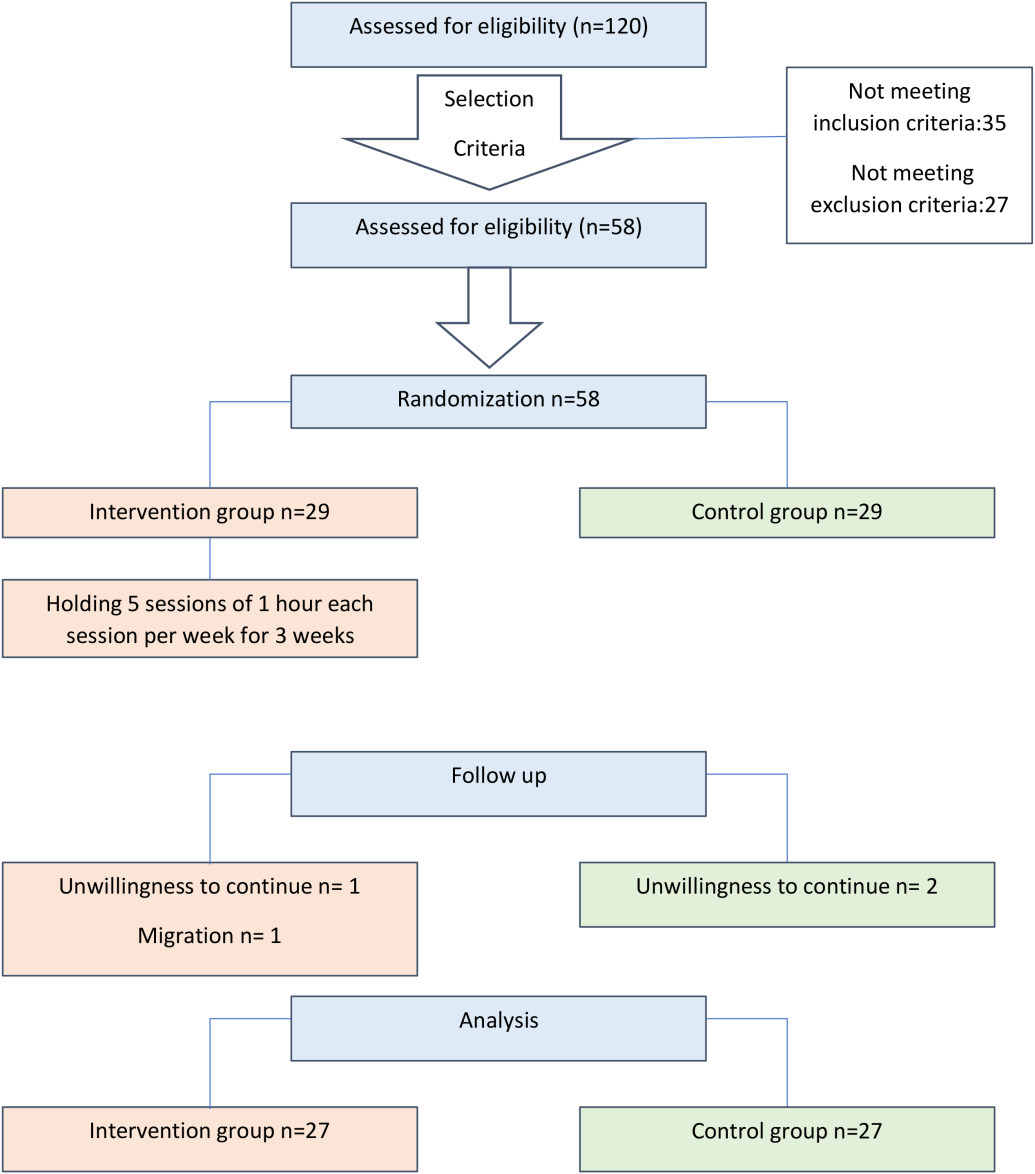
Flowchart of participants.

The patients were randomly assigned into two intervention groups VR-SIT and traditional treatment (TT), using computer-generated randomization. Randomization was based on a 1: 1 ratio using a computer random number generator (https://www.random.org/randomness/) that generates randomness via atmospheric noise, conducted by an independent researcher. The allocation order was kept confidential and was not available to any participant or researcher. Similarly, the statistical analyzer was not informed about the coding of the groups. The flowchart of participants is described in **Figure 1**.

### Sample size calculation

An *a priori* sample size calculation was performed in accordance with established methodological recommendations that emphasize defining the primary comparison, specifying the type I error rate and desired statistical power, and transparently reporting the underlying assumptions to support reproducibility (31, 32).

Power analysis was conducted using G*Power (version 3.1.9.7), a validated and widely used software for statistical power analyses across F-test families, including ANOVA (33).

The calculation was designed to detect group differences in the primary outcome using an ANOVA framework with an alpha level of 0.05, statistical power (1−β) of 0.80, and equal allocation between groups (1:1). Given the limited availability of robust prior effect size estimates for this specific intervention and population, the calculation was based on a conservative, theory-consistent detectable effect scenario for the primary comparison (32, 34–36).

Under these prespecified assumptions, the minimum required sample size was estimated at 21 participants per group.

To mitigate the potential impact of participant attrition and preserve adequate statistical power at the analysis stage, the recruitment target was increased *a priori*, consistent with methodological guidance recommending adjustment for anticipated dropout in clinical and behavioral trials. This resulted in a target sample size of 29 participants per group (total N = 58).

As reflected in the participant flow, 58 participants were randomized. Follow-up losses were limited (intervention: n = 2; control: n = 2), resulting in 27 participants per group included in the final analysis. This final analyzed sample exceeded the minimum requirement derived from the *a priori* power calculation and supports adequate statistical sensitivity for the prespecified primary comparison, in line with recommended reporting practices for randomized trials (31, 37).

### Measurements and procedure

#### Pre-treatment comparisons

Participants were assessed at baseline for sociodemographic characteristics and clinical measures, including depression severity and motivation levels. Randomization ensured that groups were balanced with respect to these baseline measures. No statistically significant differences between groups were observed in depression severity or motivation levels prior to treatment.

The sociodemographic questionnaire included the following variables: residence, marital status, duration of illness (months), sex, and age. The primary outcome of this study was depressive symptom severity measured using the Hamilton Depression Rating Scale.

The Hamilton Depression Rating Scale was developed to assess the severity of depressive symptoms. This instrument focuses primarily on somatic and behavioral aspects of depression, with particular emphasis on vegetative, cognitive, and anxiety symptoms. Each item is scored on a scale from 0 to 2, and the total score corresponds to the sum of all item scores.

Score interpretation follows commonly used clinical ranges: 0–6 indicates no depression, 7–14 mild depression, 15–24 moderate depression, and 25–52 severe depression. In terms of reliability, the scale demonstrates strong internal consistency, with a Cronbach’s alpha of approximately 0.92. The intraclass correlation coefficient has also been reported at approximately 0.92, with inter-observer reliability around 0.85 in previous studies. Regarding validity, correlations with other depression assessment instruments, including the Montgomery-Åsberg Depression Rating Scale, the Depressive Symptomatology Inventory, and the Bech Melancholia Scale, typically range between 0.8 and 0.9.

Before initiating the intervention, all participants completed the sociodemographic questionnaire and the Hamilton Depression Rating Scale with the assistance of a trained evaluator external to the research team. At the time of this baseline evaluation, participants had not yet initiated pharmacological treatment. Antidepressant medication was prescribed during the psychiatric consultation immediately following the initial clinical assessment, in accordance with routine clinical care protocols of the Peruvian public mental health system. The Hamilton Depression Rating Scale is also routinely used within the Peruvian public health system for the clinical and epidemiological evaluation of depression.

Motivation levels were recorded weekly throughout the three-week intervention (Weeks 1–3) and once more at the end of the final training session to capture immediate post-intervention motivation. The Hamilton Depression Rating Scale (HAM-D) assessments were conducted by psychology staff from the Community Mental Health Center of San Sebastian, where participants were recruited. These clinicians were part of the routine clinical staff of the center and were not members of the research team or involved in the delivery of the interventions.

The evaluations were performed according to the standardized clinical procedures used in the Peruvian public mental health system under the guidelines of the Ministry of Health (MINSA). The assessors performed the evaluations as part of routine clinical assessment and were not involved in the study design, randomization process, or intervention implementation.

### Interventions

The intervention period lasted three weeks. Participants in the experimental and control groups received treatment at the same frequency and intensity, consisting of five sessions per week (five consecutive days). Each session had a total duration of approximately 60 minutes. The experimental group received Virtual Reality–based Stress Inoculation Training (VR-SIT), whereas the control group received standard psychological treatment. Both interventions were designed to ensure equivalent therapeutic exposure, differing only in the mode of delivery (immersive VR versus conventional non-VR format).

### Pharmacological treatment

Following the baseline clinical assessment and randomization procedures, all participants received pharmacological treatment as part of standard clinical care provided within the public mental health system in Peru. In this setting, first-line pharmacological management for Major Depressive Disorder is based on selective serotonin reuptake inhibitors, primarily sertraline or fluoxetine, with short-term benzodiazepines prescribed when clinically indicated for anxiety or sleep disturbances. Pharmacological treatment followed established public-sector clinical protocols and was prescribed and monitored by a psychiatrist. 89% of participants (n = 48) received fluoxetine, while the remaining participants (n = 6) received sertraline (2 in the intervention group and 4 in the control group). Additionally, clonazepam (0.5 mg) was prescribed on a conditional basis for short-term management of anxiety or sleep disturbances when clinically indicated by the treating psychiatrist in all the patients.

Importantly, pharmacological treatment was not manipulated as an experimental variable in this study and remained stable throughout the three-week intervention period. Although medication selection was tailored to individual clinical needs, the restricted range of pharmacological agents used in this public health context limited inter-individual variability. Randomization was therefore expected to distribute any remaining pharmacological variability evenly between the VR-SIT and Traditional Treatment (TT) groups. Accordingly, the present study was designed to evaluate the differential effects of the non-pharmacological intervention modality (VR-based versus non-VR delivery) as an adjunct to standard pharmacological care, rather than to isolate the effects of VR or psychotherapy in the absence of medication.

Experimental Group: Virtual Reality Stress Inoculation Training (VR-SIT).

Participants assigned to the experimental group completed VR-SIT sessions five days per week over three consecutive weeks. Each session followed a structured Stress Inoculation Training framework, including psychoeducation, skills acquisition and rehearsal, and application within immersive virtual environments. During each session, participants interacted with a VR serious game for approximately 60 minutes under supervision.

The VR-SIT protocol was organized into three progressive levels of difficulty, corresponding to increasing stress intensity and task complexity. These levels were implemented sequentially across the three-week intervention period and were designed to gradually enhance stress tolerance, coping skills, and perceived controllability. A session-by-session outline of both intervention conditions (Traditional Treatment and VR-SIT) across the three-week program is presented in **Table 1**.

**Table 1:** Session-by-session outline of the Traditional Treatment (TT) and Virtual Reality Stress Inoculation Training (VR-SIT) interventions.

Training sessions	Session	Traditional Treatment (TT): description of each session	VR-SIT condition: description of each session	VR Game difficulty
Week 1	Session 1	Introductory psychoeducation session led by a psychologist focusing on the relationship between stress, depressive symptoms, thoughts, and emotions.	Introductory session explaining the relationship between stress, depressive symptoms, thoughts, and emotions. Orientation to the therapeutic objectives and familiarization with the VR environment in the neutral “white room.”	VR Game: 30 minutes to identify all the game virtual patients. The game is set to easy mode with mild sounds.
	Session 2	Therapeutic accompaniment session with nursing staff, focused on supportive conversation, monitoring emotional state, and reinforcing adherence to treatment recommendations.	Guided identification of stress reactions, negative thoughts, and emotional responses. Participants practice navigation and basic interaction tasks within simple VR scenarios.	
	Session 3	Session led by a psychologist aimed at identifying stress triggers and common negative thoughts associated with depressive symptoms.	Practice recognizing emotional and cognitive responses to mild stress while interacting with virtual patients. Participants learn how to pause scenarios and request guidance to reinforce perceived controllability.	
	Session 4	Therapeutic accompaniment with nursing staff, providing emotional support and encouraging the use of basic coping strategies discussed in therapy.	Relaxation-oriented session combined with low-intensity VR exposure. Participants rehearse breathing and coping responses while completing simple virtual tasks.	
	Session 5	Consolidation session led by a psychologist reviewing stress awareness and basic coping strategies introduced during the week.	Consolidation of week-1 learning. Participants integrate stress awareness and early coping responses while interacting with simple immersive scenarios.	
Week 2	Session 1	Brief psychoeducation and review of stress management strategies led by a psychologist.	Transition to moderately demanding VR scenarios. Participants begin identifying automatic negative thoughts during more complex immersive tasks.	VR Game: 15 minutes to identify all the game virtual patients. The game is set to a moderate difficulty with high sounds, screams, and chaotic noises. The patients practice 3 times during the session.
	Session 2	Therapeutic accompaniment session with nursing staff, focused on discussing daily stressors and supporting emotional regulation.	Practice of cognitive coping strategies while interacting with virtual patients in moderately stressful environments.	
	Session 3	Psychologist-led session focused on recognizing automatic negative thoughts and discussing alternative responses to stressful situations.	Relaxation and coping rehearsal within moderately stressful VR scenarios. Participants practice maintaining emotional control during increasing task complexity.	
	Session 4	Therapeutic accompaniment with nursing staff, reinforcing coping strategies and providing supportive guidance for stress management in daily life.	Participants evaluate stress responses and apply coping strategies during immersive scenarios requiring quicker responses and emotional regulation.	
	Session 5	Consolidation session reviewing coping strategies and encouraging their application in everyday situations.	Consolidation of week-2 training through guided coping practice and performance feedback following immersive tasks.	
Week 3	Session 1	Psychologist-led session reviewing progress and discussing stress management in more challenging situations.	Transition to high-intensity immersive stress exposure. Participants apply learned coping skills in demanding VR scenarios with increased sensory load and time pressure.	VR Game: 8 minutes to identify all the game virtual patients. The game is set to extreme mode with high sounds, screams, and chaotic noises. The patients practice 5 times during the session.
	Session 2	Therapeutic accompaniment session with nursing staff, focused on emotional support and reinforcing adaptive coping behaviors.	Advanced coping practice under highly demanding VR conditions requiring sustained attention and emotional regulation.	
	Session 3	Psychologist-led session discussing strategies to manage negative thoughts and emotional reactions to stress.	Integration of relaxation, cognitive coping, and behavioral regulation during complex immersive scenarios.	
	Session 4	Therapeutic accompaniment with nursing staff, reinforcing coping strategies and monitoring emotional well-being.	Extended practice session requiring coping integration and stress management across multiple immersive tasks.	
	Session 5	Final consolidation session reviewing stress management strategies and encouraging continued use of coping skills after the program.	Final consolidation session. Participants integrate all learned coping strategies in the most demanding scenarios, followed by performance feedback and review of progress.	

### Control group

Participants in the control group received standard psychological treatment focused on stress management and emotional regulation strategies, without the use of virtual reality technology. Participants assigned to the Traditional Treatment (TT) condition received structured face-to-face psychological sessions focused on stress management and coping strategies. These sessions were delivered weekly by trained nurses and psychologies from the Community Mental Health Center and followed routine clinical practice guidelines for the management of depressive symptoms and stress-related difficulties as part of the community center program for depression, including “acompañamiento terapeutico” therapeutic support by trained nurses and psychotherapy and psychoeducation support by trained psychologists.

The TT sessions included psychoeducation about stress responses, identification of emotional triggers, discussion of coping strategies, and supportive therapeutic dialogue aimed at improving stress regulation and emotional awareness. The frequency and duration of sessions were equivalent to those of the VR-SIT condition in order to maintain comparability between groups.

Sessions were conducted at the same frequency and duration as the experimental intervention (five sessions per week, approximately 60 minutes per session). In addition, participants in this group received standard pharmacological treatment according to the severity of their clinical presentation, consistent with routine psychiatric care.

### Virtual reality serious game

The VR serious game was designed to enhance perceived controllability, a core therapeutic target of VR-SIT. Each session began in a neutral virtual “white room,” which functioned as a tutorial environment allowing participants to familiarize themselves with game mechanics and controls. This initial phase aimed to reduce novelty-related anxiety and ensure task comprehension prior to stress exposure.

Following the tutorial phase, participants entered immersive virtual scenarios characterized by chaotic and emotionally salient conditions, including environmental hazards, distressed virtual characters, and auditory stressors such as alarms and screams. Within these environments, participants were required to navigate the scene and interact with virtual patients by performing tasks such as assessing vital signs, evaluating clinical conditions, and providing first aid or triage-related actions.

Stress intensity increased progressively across intervention levels through greater sensory load, environmental complexity, and time pressure. To reinforce the sense of controllability, participants could pause the scenario at any time and receive assistance from a virtual robotic guide, which provided procedural support and guidance for patient assessment and decision-making.

**Figure 2:**
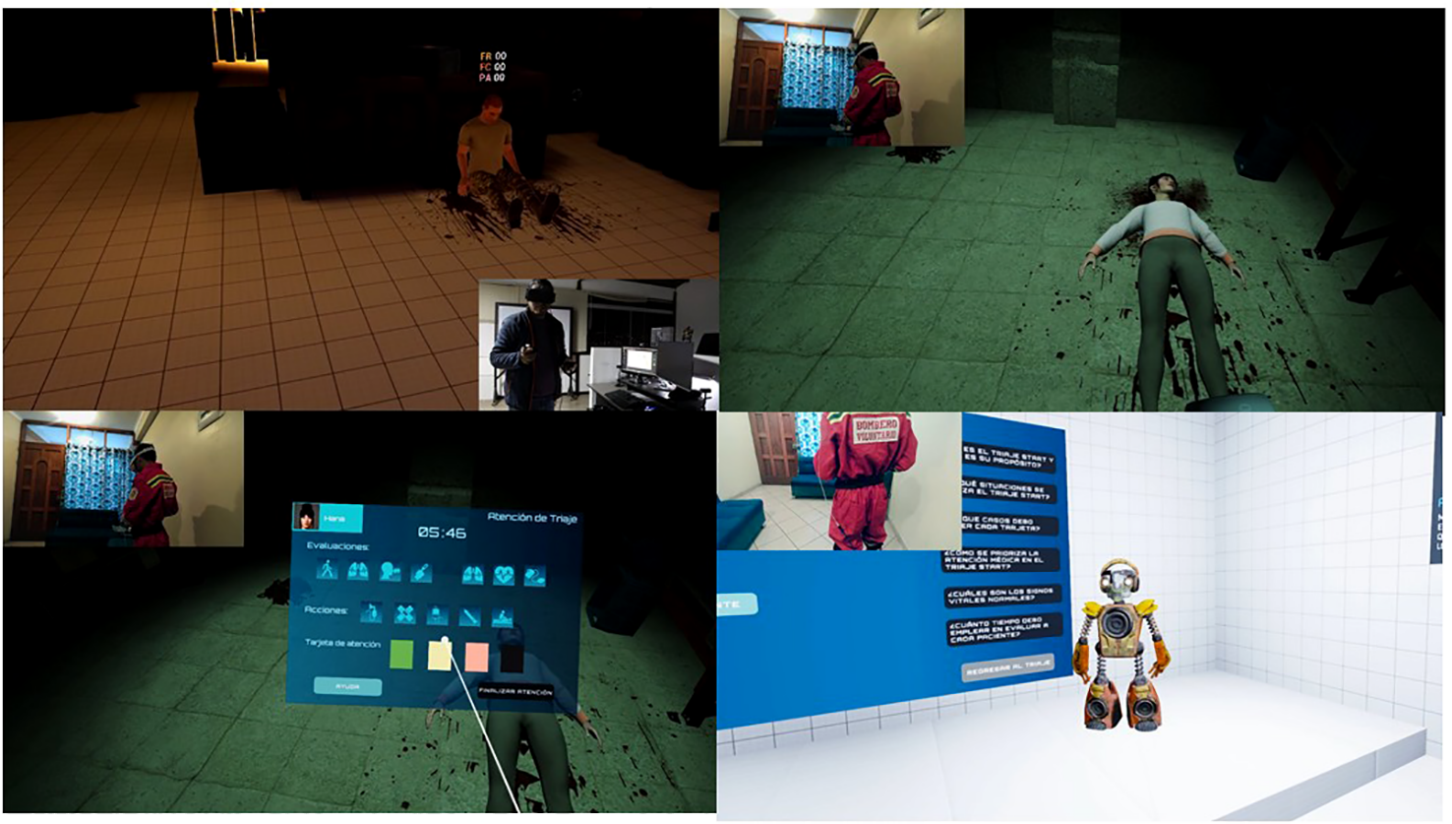
Virtual reality serious game. Left up, Starting white room; Left Down, Triage, evaluations and actions table; Right up, One patient screaming, in this case randomly generated with a hemorrhage; Right down, Another patient.

At the end of each session, participants returned to the tutorial environment, where structured performance feedback was provided, including scores, correct and incorrect actions, and the option to repeat the scenario. Virtual patients, clinical conditions, and required actions were randomly generated for each session, ensuring repeated exposure to novel stressors. Representative images of the VR serious game are presented in **Figure 2**.

### Statistical analysis

The data were analyzed using SPSS 22.0 software. Descriptive statistics, including mean, standard deviation, and frequency distributions, were calculated. A mixed-design (repeated measures) ANOVA test was performed to assess changes over time in Hamilton Depression Scale scores and motivation levels, with time as the within-subject factor and group as the between-subjects factor. *Post-hoc* comparisons were conducted using the Bonferroni correction for multiple comparisons. Effect sizes were reported as partial eta squared (η²) for repeated measures ANOVA and Cohen’s d for pairwise comparisons. The significance level was set at p < 0.05 for all tests.

## Results

### Descriptive statistics

The mean age of the participants was 26.26 years (SD = 6.71), with ages ranging from 19 to 43 years. The disease duration varied from 6 to 18 months, with a mean of 9.93 months (SD = 3.52). The Hamilton Depression Rating Scale scores before treatment had a mean of 18.15 (SD = 2.61), which decreased to 10.33 (SD = 3.50) after treatment, reflecting an overall descriptive reduction in depressive symptoms. Motivation levels showed a progressive increase, with a mean of 3.78 (SD = 1.27) in the first week, increasing to 6.11 (SD = 1.64) by the end of the training program. This progressive increase in motivation suggests an upward descriptive trend over time. **Table 2** provides an overview of the descriptive statistics for the numerical variables examined in this study.

**Table 2:** Descriptive statistics of study variables.

Variable	Mean	median	Standard deviation	Minimum	Maximum	IQR
Age	26.26	25.00	6.71	19	43	10.00
Disease Duration (months)	9.93	10.00	3.52	6	18	6.00
Hamilton Depression Rating Scale before treatment	18.15	18.00	2.61	15	23	4.00
Hamilton Depression Rating Scale after treatment	10.33	10.00	3.50	5	18	6.00
Motivation Week 1	3.78	4.00	1.27	1	7	2.00
Motivation Week 2	5.00	5.00	1.24	2	8	2.00
Motivation Week 3	5.89	6.00	1.58	2	9	2.00
Motivation at the end of the training	6.11	6.00	1.64	2	10	2.00

The majority of participants resided in Cusco (66.67%), followed by Urubamba (11.11%), Calca (9.26%), Quillabamba (7.41%), and other locations (5.56%). The marital status of participants was predominantly single (87.04%), with fewer participants being married (9.26%) or widowed (3.70%). The gender distribution showed a higher percentage of female participants (77.78%) compared to male participants (22.22%). This demographic profile highlights the sample characteristics, indicating a higher representation from Cusco and a predominantly single and female participant group as is seen in **Table 3**.

**Table 3:** Frequency distribution of categorical variables.

Residence	Frequency	Percentage (%)
Cusco	36	66.67
Urubamba	6	11.11
Calca	5	9.26
Quillabamba	4	7.41
Other	3	5.56
Marital Status	Frequency	Percentage
Single	47	87.04%
Married	5	9.26%
Widowed	2	3.70%
Gender	Frequency	Percentage
Female	42	77.78%
Male	12	22.22%

### Correlation analysis

**Table 4** presents the correlations between the main study variables. A negative, non-significant correlation was observed between age and disease duration (r = -0.235), but this association did not reach statistical significance (p = 0.098). Motivation scores across consecutive weeks were strongly and positively correlated, particularly between Week 3 and the end of training (r = 0.954, p < 0.001), indicating temporal consistency in motivation levels. Age was significantly negatively correlated with motivation at the end of training (r = -0.293, p = 0.031). The remaining correlations, including those involving disease duration, post-treatment Hamilton Depression Rating Scale scores, and earlier weekly motivation scores, were weak and did not reach statistical significance.

**Table 4:** Correlation coefficients (r) and p-values for the relationships between study variables.

Variables	Correlation (r)	p-value
Age and Disease Duration	-0.235	0.098
Hamilton Depression Rating Scale before and after treatment	0.109	0.433
Motivation Week 1 and Week 2	**0.762**	**<0.001**
Motivation Week 2 and Week 3	**0.896**	**<0.001**
Motivation Week 3 and End of Training	**0.954**	**<0.001**
Age and Motivation at the end of the training	**-0.293**	**0.031**
Age and Motivation Week 1	-0.096	0.495
Age and Motivation Week 2	-0.116	0.406
Age and Motivation Week 3	-0.159	0.237
Age and Hamilton Depression Rating Scale after treatment	0.107	0.419
Disease Duration and Hamilton Depression Rating Scale after treatment	-0.091	0.529
Disease Duration and Motivation Week 1	-0.111	0.458
Disease Duration and Motivation Week 2	0.023	0.861
Disease Duration and Motivation Week 3	0.041	0.760
Disease Duration and Motivation End of Training	0.079	0.588

Bold are statistical significant values.

### Repeated measures ANOVA analysis

A repeated measures ANOVA was conducted to analyze changes in Hamilton Depression Scale scores and motivation levels, with time as the within-subject factor and treatment condition (VR-SIT vs. Traditional Treatment) as the between-subjects factor. *Post-hoc* Bonferroni corrections were applied to account for multiple comparisons, and effect sizes were reported using partial eta squared (η2). The significance threshold was set at p < 0.05.

The main effect of treatment condition was statistically significant, F(1,52)=4.36, p=0.042, η2 = 0.10. However, Bonferroni-adjusted pairwise comparisons did not show significant between-group differences at post-treatment (p = 0.753, d = 0.07), indicating that the observed omnibus effect should be interpreted cautiously and may reflect baseline variability rather than a clinically meaningful treatment difference. The time-by-group interaction was small-to-moderate, F(1,52)=4.98, p=0.031, η2 = 0.10, suggesting some variation over time. *Post-hoc* Bonferroni comparisons for depression scores confirmed no significant differences between groups at any time point (Pre: p = 0.842, Post: p = 0.753).

For motivation levels, a significant main effect of time was detected, F(3,162)=7.89, p<0.001, η2 = 0.15, indicating an overall increase in motivation levels across all participants. The main effect of treatment condition was also significant, F(1,52)=6.23, p=0.018, η2 = 0.12, with VR-SIT participants demonstrating greater motivation scores. A significant time-by-group interaction, F(3,162)=7.02, p=0.011, η2 = 0.13, confirmed that motivation increased more prominently in the VR-SIT condition. *Post-hoc* Bonferroni-adjusted comparisons confirmed that motivation levels did not significantly differ between groups at Week 1 (p=0.094), indicating similar baseline motivation levels. However, significant between-group differences emerged at Week 2 (p=0.007), Week 3 (p=0.004), and post-treatment (p=0.001), with VR-SIT participants demonstrating greater motivation improvements over time.

The moderate-to-large effect sizes (η2 = 0.12−0.15) indicate that the improvement in motivation was not only statistically significant but also substantial in practical terms, favoring VR-SIT over Traditional Treatment.

Mauchly’s test for sphericity did not indicate violations (p > 0.05), confirming the validity of repeated measures ANOVA assumptions. Shapiro-Wilk tests confirmed normally distributed residuals (p > 0.05), and Levene’s test indicated homogeneity of variance across groups (p > 0.05). Outliers were assessed using the interquartile range (IQR) method, and no extreme values were identified. The absence of floor or ceiling effects was confirmed by the gradual and continuous increase in motivation scores without clustering at scale extremes. This is observed in the **Tables 5**, **6**.

**Table 5:** Summary of repeated measures ANOVA results.

Factor	F	p-value	Partial η2
Time (Depression)	4.85	0.032	0.08
Group (Depression)	4.36	0.042	0.10
Time × Group (Depression)	4.98	0.031	0.10
Time (Motivation)	7.89	<0.001	0.15
Group (Motivation)	6.23	0.018	0.12
Time × Group (Motivation)	7.02	0.011	0.13

**Table 6:** Descriptive statistics for depression and motivation scores.

Measure	Time	VR-SIT (M ± SD)	Traditional (M ± SD)
Depression	Pre	18.32 ± 2.58	17.91 ± 2.61
	Post	9.61 ± 3.38	10.88 ± 3.52
Motivation	Week 1	4.07 ± 1.23	3.75 ± 1.26
	Week 2	5.81 ± 1.21	4.79 ± 1.24
	Week 3	6.88 ± 1.49	5.38 ± 1.57
	End	7.49 ± 1.53	5.79 ± 1.63

## Discussion

Building on the theoretical framework outlined in the Introduction, emphasizing stress regulation, perceived controllability, and engagement as key mechanisms relevant to Major Depressive Disorder (MDD), the main objective of this study was to evaluate the potential effectiveness of Virtual Reality (VR) Stress Inoculation Training (SIT) in reducing depressive symptoms and enhancing motivation among individuals with mild to moderate depression, compared with a traditional therapeutic approach. Both VR-SIT and Traditional Treatment led to significant reductions in depression scores over time, with no significant between-group differences at any time point. For motivation, initial levels were comparable between groups at Week 1 (p = 0.094), but VR-SIT demonstrated significantly greater increases at Week 2 (p = 0.007), Week 3 (p = 0.004), and post-treatment (p = 0.001). The moderate-to-large effect sizes suggest that VR-SIT provided a sustained advantage in engagement and motivation beyond Traditional Treatment. This pattern suggests that VR-SIT may represent a promising modality for motivational engagement, whereas short-term symptom reduction may be comparable to an active control condition receiving standard pharmacotherapy and psychotherapy.

The absence of between-group differences in depressive symptom reduction is consistent with evidence that multiple structured interventions, particularly when combined with routine pharmacological management, can produce meaningful short-term symptom improvement. For example, internet-based and self-guided cognitive behavioral interventions have demonstrated significant reductions in depressive symptoms, and meta-analytic evidence supports robust antidepressant effects across a broad range of psychotherapies (38). Within this landscape, the present findings do not indicate that VR-SIT lacks antidepressant activity; rather, they suggest that VR-SIT may not exceed the symptom-reducing effects of an active, clinically credible comparator delivered with similar intensity. This interpretation is strengthened by the short intervention duration (three weeks), which may be sufficient to observe early symptom improvement in both groups but insufficient to detect divergence between modalities, particularly in mild to moderate cases.

The motivation results are consistent with theoretical and empirical work indicating that immersion, interactivity, and agency can strengthen engagement-related outcomes in psychological interventions. VR-based approaches provide controlled, ecologically valid scenarios that increase emotional salience and active participation (13, 14), and serious-game mechanics may further support adherence and motivation through immediate feedback, goal structure, and experiential learning (12). In the present protocol, the game design emphasized perceived controllability—allowing participants to pause the scenario and obtain guided assistance—features that may counter learned helplessness and strengthen mastery experiences, processes closely linked to motivation in depression (14). Accordingly, the observed motivational advantage aligns with the conceptual rationale for controllability-focused VR interventions and the broader claim that VR can enhance emotional engagement and cognitive-behavioral learning processes (25–27).

From a psychotherapy-model perspective, the VR-SIT intervention can be interpreted as an adaptation of Meichenbaum’s Stress Inoculation Training framework, which emphasizes conceptualization, coping skill rehearsal, and application under progressively challenging conditions (15, 16). The three-level structure implemented sequentially across weeks constitutes a graded exposure and skills-application format consistent with SIT principles (15), and similar structured or digital SIT adaptations have demonstrated feasibility and symptom-related benefits across diverse contexts (17–21). The present results extend this literature by suggesting that embedding SIT-consistent coping practice within immersive, controllability-enhancing VR scenarios may preferentially influence motivation and engagement during treatment. This aligns with prior conceptual arguments that VR-based SIT may improve generalization of coping skills by allowing repeated rehearsal in realistic yet safe conditions (22), including graded stress exposure approaches used in other stress-related clinical domains (23).

When comparing these results to prior VR interventions in depression, the current pattern is also consistent with evidence that VR may function primarily as an engagement-enhancing delivery modality rather than a uniformly superior antidepressant intervention. VR has shown promise across exposure-based, CBT-informed, and mindfulness-oriented approaches for depression (25–27), and multiple studies report clinically meaningful improvements in depressive symptoms and well-being (28–30). However, comparative trials have not consistently demonstrated superiority of VR over high-quality traditional psychotherapies. In line with this, Meyerbröker et al. reported no significant differences in depressive symptom reduction between VR-based and traditional CBT approaches (39). The present study complements these findings by suggesting that even when symptom reduction is comparable, VR-based delivery—particularly with explicit controllability and feedback components—may yield measurable advantages in motivational trajectories during treatment.

Several design features may have reduced the likelihood of detecting between-group differences in depressive severity while still allowing differences in motivation to emerge. The TT condition included standard pharmacotherapy and psychotherapy but likely involved heterogeneity in therapeutic techniques, which could dilute contrasts with VR-SIT and increase within-group variance in symptom outcomes. Additionally, depressive symptom change may require longer follow-up to detect downstream effects mediated by improved motivation and engagement, particularly if early motivational gains translate into better adherence, behavioral activation, or sustained treatment participation over time. Taken together, these considerations support interpreting VR-SIT as a promising adjunctive strategy for engagement and motivation in MDD, while highlighting the need for future trials with standardized comparators and longer-term follow-up to determine whether motivational advantages produce delayed or sustained symptom benefits.

In addition, Freeman et al. reported that immersive VR interventions were associated with increased emotional engagement and perceived control, while depressive symptom changes were comparable to those observed with conventional treatment approaches (13). Falconer et al. similarly observed improvements in self-referential processing and engagement following VR-supported cognitive interventions in patients with depression, without consistent superiority over standard therapies in reducing depressive symptoms (30). Lindner et al. reported high levels of acceptability and familiarity with VR-based therapeutic approaches among practicing cognitive behavioral therapists, highlighting the growing integration of immersive technologies into psychological treatment contexts (40). Meta-analytic findings indicate that VR-based psychological interventions produce moderate effects on depressive symptoms that are broadly comparable to established psychotherapeutic approaches, while also facilitating experiential learning and treatment adherence (26). Furthermore, Riva et al. described that VR interventions emphasizing controllability, agency, and graded exposure may primarily influence motivational and self-efficacy processes, which may contribute to treatment engagement and longer-term outcomes rather than immediate changes in symptom severity (41). Taken together, these findings provide context for the present results, suggesting that VR-SIT may influence motivational dimensions of treatment response while producing depressive symptom changes comparable to those observed with standard interventions.

### Limitations

One of the primary limitations is the absence of follow-up evaluations to determine the long-term effects of the intervention. While the immediate outcomes demonstrated significant reductions in depressive symptoms and increases in motivation, the sustainability of these improvements remains uncertain. Long-term follow-up assessments at intervals such as three- or six-months post-intervention would provide more robust insights into the enduring impact of the intervention. To partially address this, we ensured rigorous documentation of the intervention process and participant adherence, which serves as a foundation for future studies incorporating follow-up designs. Although the control group received comparable psychological treatment, the variability in therapeutic techniques and their application might have influenced the comparability of outcomes. To mitigate this issue, we ensured that the frequency of psychological sessions in the control group matched the experimental group and documented the general characteristics of the therapies provided. However, future research should aim to standardize and comprehensively describe the control group interventions to enable more precise comparisons. Additionally, the study’s sample size, while adequate for detecting significant differences, limits the generalizability of the findings to larger and more diverse populations. Self-reported measures were primarily used to assess depressive symptoms and motivation, introducing potential biases such as social desirability or inaccuracies in participant reporting. To minimize these effects, validated and widely recognized scales were employed, and participants were provided with clear instructions to encourage honest responses. Nevertheless, future research could benefit from incorporating objective measures or clinician-rated assessments to complement self-reported data and enhance the reliability of the findings.

Lastly, while the study demonstrates the efficacy of VR-SIT, it is important to note that the intervention was delivered under supervised, standardized clinical conditions. In this context, the term “controlled clinical environment” refers to structured delivery, monitoring of adherence, and reduced contextual variability across sessions, rather than to a non-clinical, self-administered, or remotely delivered intervention format. The feasibility and effectiveness of implementing VR-SIT in real-world settings with less standardized delivery and greater contextual variability remain to be evaluated. We attempted to address this by designing the VR intervention to be adaptable to various settings, but further research is required to assess its practical application outside of clinical trials.

Although Stress Inoculation Training (SIT) is not considered a first-line treatment for Major Depressive Disorder, it is increasingly recognized as a relevant adjunctive intervention when stress dysregulation, perceived helplessness, and reduced coping capacity contribute to the maintenance of depressive symptoms. In the present study, SIT was not conceptualized as a replacement for pharmacological treatment, but rather as a complementary, non-pharmacological strategy targeting stress-related mechanisms that are not directly addressed by antidepressant medication. Pharmacological treatment primarily modulates neurochemical pathways associated with mood regulation, whereas SIT focuses on enhancing coping skills, perceived controllability, and stress appraisal, which are closely linked to motivational deficits and functional impairment in MDD. The emphasis on stress regulation also informed the design of the virtual intervention, as immersive and controllability-oriented VR environments were specifically intended to facilitate experiential learning and engagement with stress-coping skills. This design enabled the evaluation of VR-SIT as an adjunctive enhancement rather than as an alternative to standard pharmacological care.

One important limitation of the present study is that the experimental protocol and analysis plan were not preregistered in an international public preregistration platform prior to data collection. Preregistration is widely recommended to enhance transparency and to distinguish confirmatory from exploratory analyses. Future studies should address this limitation by complementing institutional approval processes with public preregistration.

Nevertheless, the study protocol was formally registered and approved prior to study initiation in the institutional research database of the Universidad Andina del Cusco (Decree No. 02-2023-DGI-UAC; University Resolution No. 260-CU-2023-UAC), following administrative, scientific, and budgetary evaluation. The study also received favorable ethical approval from the Institutional Research Ethics Committee of the Universidad Andina del Cusco (Ethics Evaluation Report No. 026-2024-CIEI-UAC; Project Code: 02024-CIEI), which reviewed the scientific rationale, sample size, participant recruitment, data confidentiality, and informed consent procedures.

An additional limitation concerns the characterization of pharmacological treatment. Although medication remained stable during the three-week intervention period according to clinical records and no medication changes were introduced as part of the study protocol, the exact duration of antidepressant treatment prior to enrollment was not systematically recorded. Because some participants were recruited shortly after their initial psychiatric evaluation, it cannot be fully excluded that early-stage pharmacological effects may have contributed to part of the observed clinical improvement.

Furthermore, although antidepressant treatment consisted primarily of selective serotonin reuptake inhibitors, the distribution of medications was not balanced across drug types. The majority of participants (approximately 89%) received fluoxetine, whereas only six participants received sertraline. Due to this limited number of sertraline-treated patients, subgroup analyses by antidepressant type were not statistically feasible. In addition, the study did not include systematic longitudinal monitoring of medication adjustments or adherence beyond confirming that no medication switches or dose changes occurred during the intervention period.

Future studies should incorporate more detailed pharmacological documentation, including duration of treatment prior to enrollment, dosage, adherence monitoring, and balanced distribution of medication types, in order to further minimize potential pharmacological confounding effects.

Although outcome assessments were performed by independent psychology staff from the Community Mental Health Center and not by members of the research team, formal blinding of assessors to treatment allocation could not be fully guaranteed within the clinical setting. Future studies should incorporate fully blinded outcome assessors to further minimize potential measurement bias.

Another limitation concerns the measurement of motivation. In the present study, motivation was assessed using a single-item visual analog scale (VAS). While this method allowed practical and repeated assessment during the intervention period, single-item measures are inherently limited and do not represent validated multidimensional instruments for assessing motivational constructs. Therefore, the results should be interpreted cautiously, as the measure may capture broader aspects of behavioral activation or engagement rather than a specific construct of treatment motivation.

### Practical implications

The results of the present study suggest that Virtual Reality Stress Inoculation Training (VR-SIT) may have practical relevance as an adjunctive intervention in the management of mild to moderate major depressive disorder, particularly with respect to motivational and engagement-related outcomes. While reductions in depressive symptom severity were comparable between VR-SIT and Traditional Treatment, the greater and progressive increase in motivation observed in the VR-SIT group suggests that this approach may be useful in clinical contexts where low motivation and limited engagement represent significant barriers to treatment participation.

From an implementation perspective, the structured and protocolized nature of the VR-SIT intervention may facilitate standardization of therapeutic delivery across clinicians and settings, potentially reducing variability associated with non-manualized psychological interventions. The use of immersive, controllability-focused virtual environments provides an experiential format for practicing coping strategies under graded stress conditions, which may be particularly relevant for patients who experience difficulties engaging with predominantly verbal or abstract therapeutic approaches.

### Future research directions

Future research should focus on exploring the long-term effects of Virtual Reality Stress Inoculation Training (VR-SIT) and its comparative efficacy with traditional psychotherapeutic approaches in diverse populations. Longitudinal studies with follow-up assessments are essential to determine the sustainability of both motivational improvements and reductions in depressive symptoms over extended periods. Moreover, investigating the underlying mechanisms of VR-SIT, particularly its role in enhancing motivation and engagement through immersive and interactive environments, will provide critical insights into its unique therapeutic advantages. Future work should also aim to standardize VR-SIT protocols, including the integration of Stress Inoculation Training phases with tailored virtual environments, to ensure consistent implementation and improve comparability across studies. Finally, expanding research to include other clinical populations, such as individuals with anxiety disorders or chronic stress, and assessing the cost-effectiveness of VR-SIT in routine clinical practice will be instrumental in broadening its applicability and accessibility.

## Conclusion

In conclusion, the study indicates that both traditional treatment and VR-SIT were associated with reductions in depressive symptoms over the intervention period, as evidenced by significant decreases in Hamilton Depression Rating Scale scores. Both groups also exhibited a progressive increase in motivation levels throughout the intervention period. Notably, no significant differences were observed between the VR-SIT and traditional treatment groups in terms of depressive symptom reduction, suggesting comparable efficacy in managing these symptoms. However, significant differences were identified in motivation levels, particularly during Week 3 and at the end of the training, with the VR-SIT group showing superior outcomes. This finding highlights the potential of VR-SIT to enhance patient motivation, an important factor in treatment adherence and overall therapeutic success. The average age of participants was 26.26 years, and the mean disease duration was 9.93 months. In addition, age showed a modest negative association with motivation at the end of training. The repeated increase in motivation scores across the intervention period suggests a consistent pattern of motivational improvement, particularly in the VR-SIT group. These findings align with existing literature on the efficacy of both VR-based and traditional psychotherapeutic interventions in managing depressive symptoms and highlight VR’s unique advantages in fostering motivational engagement.

## Data Availability

The raw data supporting the conclusions of this article will be made available by the authors, without undue reservation.
